# A pure intertubular testicular seminoma mimicking a burned-out tumor: a case report

**DOI:** 10.3389/fonc.2026.1814074

**Published:** 2026-05-15

**Authors:** Vassiliki Pasoglou, Sandy Van Nieuwenhove, Hélène Dano, Brieuc Sautois, Laurence Rocher, Julien Van Damme, Bertrand Tombal, Emmanuel Seront

**Affiliations:** 1 Department of Radiology, Cliniques Universitaires Saint-Luc, Brussels, Belgium; 2 Department of Pathology, Cliniques Universitaires Saint-Luc, Brussels, Belgium; 3 Department of Medical Oncology, Centre Hospitalier Universitaire (CHU) Sart-Tilman, Liège, Belgium; 4 Department of Radiology, Hôpital Antoine-Béclère, Université Paris-Saclay, Paris, France; 5 Department of Urology, Cliniques Universitaires Saint-Luc, Brussels, Belgium; 6 Department of Medical Oncology, Cliniques Universitaires Saint-Luc, Brussels, Belgium

**Keywords:** burned-out tumor, multiparametric MRI, pure intertubular seminoma, scrotal ultrasound, seminoma

## Abstract

**Background:**

In men presenting with metastatic seminoma and no discrete intratesticular mass, multiparametric MRI (mpMRI) findings such as atrophy, heterogeneity, calcifications, and focal low-signal areas are often interpreted as a regressed (“burned-out”) testicular primary. Rare non–mass-forming patterns, however, may produce similar indirect features and complicate primary-site attribution.

**Case presentation:**

A 37-year-old man presented with abdominal pain and a 7-8-cm retroperitoneal mass; biopsy confirmed seminoma. Scrotal ultrasound demonstrated right testicular atrophy with diffuse parenchymal heterogeneity and a punctate calcification, without a focal lesion. Testicular mpMRI showed marked heterogeneity with a poorly defined subcapsular T2-hypointense pseudonodular area, heterogeneous enhancement with focal relative hypoperfusion, and regions of increased apparent diffusion coefficient—an appearance considered compatible with a burned-out tumor. Right inguinal orchiectomy performed prior to systemic therapy revealed diffuse viable seminoma with an exclusive intertubular growth pattern, associated germ cell neoplasia *in situ*, and prominent fibro-sclerotic remodeling, without a macroscopic mass. After three cycles of chemotherapy, the patient achieved a complete metabolic response.

**Conclusion:**

This radiologic–pathologic correlation illustrates that diffuse viable intertubular seminoma with fibro-sclerotic remodeling can mimic burned-out tumor on mpMRI. In metastatic seminoma, subtle ipsilateral testicular abnormalities on ultrasound/mpMRI should prompt orchiectomy to secure definitive primary-site pathology and avoid misclassification as regression or extragonadal disease.

## Introduction

Seminoma is the most common testicular germ cell tumor, accounting for approximately 50% of germ cell tumors. It typically affects young men and presents as a well-circumscribed solid intratesticular nodule with fibrous septa and lymphocytic infiltrates, arising from germ cell neoplasia *in situ* (GCNIS) (1). Rarely, seminoma exhibits an exclusive intertubular growth pattern, referred to as pure intertubular seminoma (PITS), in which tumor cells permeate intertubular spaces without forming a discrete mass (2). On scrotal ultrasound (US) and multiparametric magnetic resonance imaging (mpMRI), PITS may manifest only as testicular atrophy and parenchymal heterogeneity, features that can be falsely reassuring or misclassified as a burned-out tumor (BOT) or even suggest an extragonadal primary (3–5).

BOT is uncommon (accounting for approximately 2–10% of germ cell tumors) and denotes spontaneous regression of a testicular primary with or without metastases; it remains a pathologic diagnosis that requires histologic evidence of intratesticular regression with absent or only minimal residual viable tumor. Proposed mechanisms include ischemic and immune-mediated regression (6–8). Because imaging features suggestive of regression do not exclude viable neoplasia, orchiectomy remains necessary to establish the diagnosis.

The present case underscores this diagnostic pitfall: imaging findings considered compatible with BOT ultimately concealed an occult, clinically significant seminoma characterized by a diffuse intertubular growth pattern.

### Case description

In October 2025, a 37-year-old man presented with a 3-month history of abdominal pain, initially intermittent and dull, then progressively increasing in frequency and intensity until it became continuous. The pain was associated with nausea and reduced appetite. An abdominal computed tomography (CT) performed in the emergency department revealed a large and heterogeneous retroperitoneal anterocaval and periduodenal mass, measuring up to 7 cm, with enhancing solid components and non-enhancing cystic areas (**Figure 1A**). Laboratory tests, including β-human chorionic gonadotropin (β-HCG) and alpha foetoprotein (AFP) were unremarkable except for mildly elevated lactate dehydrogenase (246 U/L).

**Figure 1:**
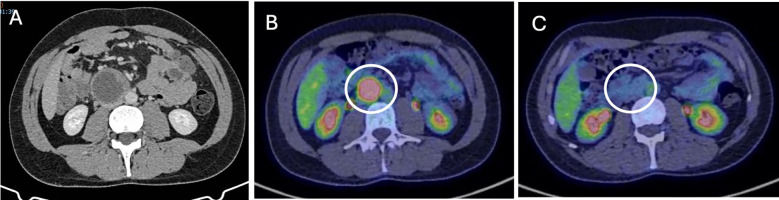
**(A)** Axial abdominal computed tomography at initial presentation describing a large and heterogeneous retroperitoneal anterocaval and periduodenal mass, measuring up to 7 cm, with enhancing solid components and non-enhancing cystic areas. **(B)** Pre-chemotherapy axial fused FDG PET-CT images demonstrating marked FDG uptake in the lesion measuring 7cm x 3cm (White circle). **(C)** Post-chemotherapy Axial fused FDG PET-CT images (8 weeks after the end of chemotherapy) showing complete disappearance of pathologic uptake within the residual lesion (4.5cm x 1.8cm), consistent with complete metabolic response (white circle).

Endoscopic US demonstrated a heterogeneous solid retroperitoneal mass adjacent to the second portion of the duodenum, measuring at least 80 mm, with a central necrotic component in the largest lobulation. The lesion was hypovascular on Doppler and contrast-enhanced ultrasound and abutted the inferior vena cava, aorta, and duodenal wall without signs of invasion. The US–guided fine needle biopsy demonstrated seminomatous tumor cells arranged in small clusters, composed of large polygonal cells with abundant clear cytoplasm and prominent central nuclei, with focal necrosis (**Figure 2A**); OCT3/4 immunostaining was positive in the tumor cells, confirming the diagnosis of seminoma (**Figure 2B**). Thoracic CT and brain MRI showed no visceral or intracranial metastases. The patient had no significant past medical history. In particular, he reported no history of scrotal trauma, cryptorchidism, orchidopexy, infertility, or chronic testicular inflammatory condition. He had fathered two children, the youngest born 6 months before diagnosis.

**Figure 2:**
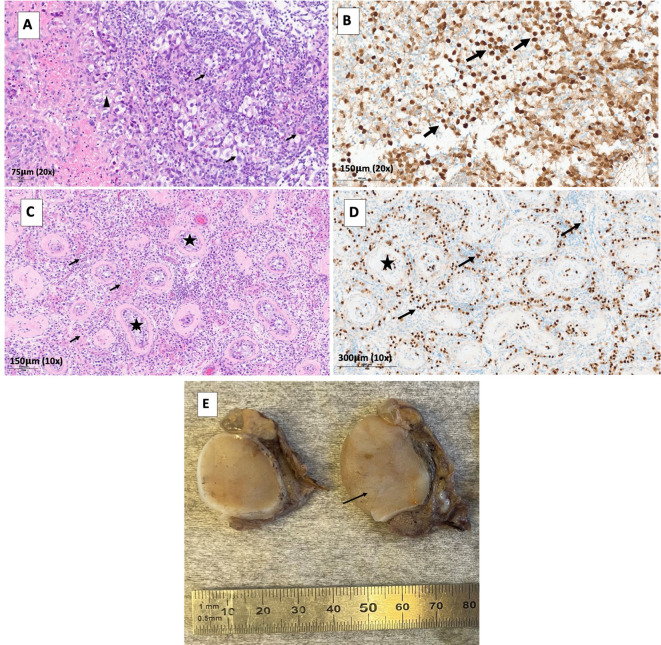
**(A)** Retroperitoneal biopsy with hematoxylin and eosin stain (Gx20): Seminomatous tumor proliferation is arranged in small clusters, consisting of large polygonal cells with abundant, clear cytoplasm and a large central nucleus (arrows). Presence of focal tumor necrosis (triangle). **(B)** Retroperitoneal biopsy: OCT3/4 immunostain (Gx20) marks the tumor cells (arrows). **(C)** Orchidectomy: The seminomatous cells are arranged in rows or in small clusters and occupy the interstitium, wrapping around the seminiferous tubules without destroying them (arrows). Most of the seminiferous tubules are atrophic, with thickened, sclerotic walls (stars). Malignant germ cells are found within their lumen (hematoxylin and eosin stain, Gx10). **(D)** Orchidectomy: Immunostain for OCT3/4 (Gx10) highlights seminomatous cells infiltrating the interstitial tissue between the seminiferous tubules (arrows) and malignant germ cells in their lumen (stars). **(E)** Orchidectomy: Macroscopical view: testicular parenchyma appears homogeneous with no visible mass and has a near-normal beige color (arrow).

A scrotal US (**Figures 3A, B**) showed right testicular atrophy (3.0 x 1.8 x 2.6cm compared to 4.0 x 2.0 x 2.8 cm for the left testis) with diffuse parenchymal heterogeneity and architectural distortion, without a discrete intratesticular mass. A punctate intraparenchymal calcification was also noted, while Doppler imaging showed preserved vascularity without focal hypervascularization. Based on these anomalies, a scrotal mpMRI (**Figures 3C–F**) was performed and described marked parenchymal heterogeneity, with a poorly defined subcapsular T2-hypointense pseudonodular region associated with hypoperfused areas rather than a well-circumscribed intratesticular nodule (**Figures 3C–E**). Diffusion-weighted imaging demonstrated increased apparent diffusion coefficient (ADC) values (approximately 2000 × 10^-6^ mm²/s), consistent with low cellularity related to fibrosis or tumor regression (**Figure 3F**). These imaging findings were considered compatible with a BOT with probable focal residual viable disease.

**Figure 3:**
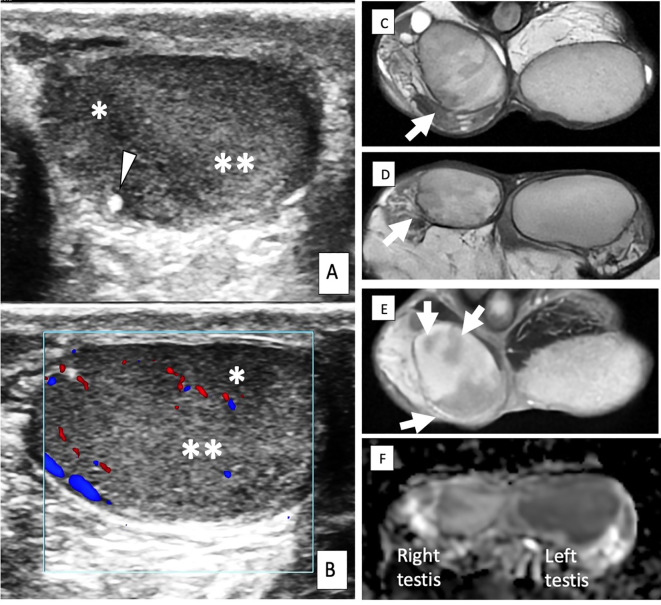
**(A)** Grayscale and **(B)** color Doppler Scrotal ultrasound demonstrate right testicular atrophy with diffuse parenchymal heterogeneity (hypoechoic * and hyperechoic **) and architectural distortion, without evidence of a discrete intratesticular mass. A punctate intraparenchymal echogenic focus consistent with calcification is present (arrowhead in A). Color Doppler imaging shows preserved intratesticular vascularity without focal hypervascularization. **(C-F)** Multiparametric scrotal magnetic resonance imaging: Coronal T2-weighted **(C)**, axial T2-weighted **(D)**, coronal fat-suppressed contrast-enhanced T1-weighted **(E)**, and ADC map **(F)**. The right testis shows marked parenchymal heterogeneity with a poorly defined subcapsular T2-hypointense area (arrows in C and D), heterogeneous enhancement with focal hypoperfused areas (arrows in E), and heterogeneous ADC **(F)** map including areas with high ADC values (1940 × 10^-6^ mm²/s) compared with the left testis (1050 × 10^-6^ mm²/s), without a well-circumscribed intratesticular mass.

### Timeline

Despite suggesting an underlying testicular abnormality, the absence of definitive imaging features precluded a confident diagnosis. Because biopsy of the retroperitoneal mass had established seminoma, the principal radiologic differential diagnosis in this case was whether the ipsilateral testicular abnormality represented a BOT or an occult viable seminomatous primary with a non–mass-forming pattern such as PITS. BOT usually suggests regression of a primary germ cell tumor and may be associated with a fibrotic, calcified, or scar-like testicular abnormality, whereas PITS corresponds to viable seminoma infiltrating the testicular parenchyma without forming a distinct mass. Other less likely imaging differentials include segmental infarction or hematoma, inflammatory or granulomatous orchitis, and testicular lymphoma. Segmental infarction or hematoma generally shows absent or markedly reduced vascularity. Orchitis more often presents with pain, inflammatory symptoms, and hyperemic or diffuse epididymo-testicular changes. Lymphoma may also manifest as diffuse, non–mass-like testicular infiltration, but it usually shows pronounced hypervascularity or bilateral disease. Nevertheless, imaging overlap remains substantial, and definitive diagnosis requires pathological examination after orchiectomy. Right inguinal orchiectomy was therefore performed before starting systemic therapy (**Figure 4**).

**Figure 4:**
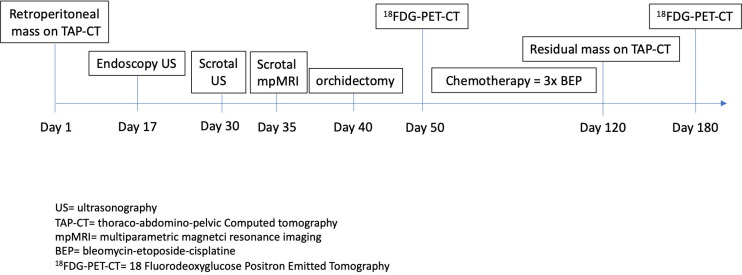
Timeline.

### Diagnostic assessment

Histological examination showed diffuse involvement by pure seminoma with an intertubular growth pattern, prominent fibro-sclerotic remodeling (approximately 40%) and adjacent GCNIS (**Figures 2C, D**). Focal invasion of the rete testis, tunica albuginea, and hilar soft tissue was present (pT2, R0), without lymphovascular invasion or spermatic cord involvement. Immunohistochemistry confirmed seminoma (OCT3/4+, CD117+) with no evidence of non-seminomatous elements (**Figures 2C, D**). Overall, these findings supported a diagnosis of PITS with diffuse testicular infiltration. Importantly, gross examination revealed normal, homogeneous beige parenchyma without a visible mass (**Figure 2E**).

The final stage was pT2 cN3 cM0 S0/S1, corresponding to stage IIC seminoma. In accordance with guideline-recommended first-line treatment for good-risk metastatic seminoma, the patient received three cycles of bleomycin, etoposide, and cisplatin (BEP) chemotherapy, with etoposide and cisplatin administered on days 1–5 and bleomycin on days 2, 8, and 15 of each 21-day cycle (9). Before chemotherapy initiation, ^18^Fluorodeoxyglucose (FDG) positron emission tomography-computed tomography (PET-CT) was performed to assess the metabolic activity of the retroperitoneal lesion. It demonstrated intense uptake in the retroperitoneal mass (7cm x 3cm) and no other suspicious lesions (**Figure 1B**). Eight weeks after the end of chemotherapy, in April 2026, the ^18^FDG PET-CT showed a decrease in the retroperitoneal size (4.6 cm x 1.8 cm) and a complete disappearance of the tracer uptake, consistent with a complete metabolic response (**Figure 1C**). Laboratory tests, including LDH, β-HCG and AFP were within normal ranges. The patient subsequently entered surveillance follow-up. Although routine serial US of the contralateral testis is not guideline-mandated, the unusual non–mass-forming presentation in this case supports further careful clinical surveillance, with a low threshold for repeat scrotal US if any abnormality is suspected (9).

## Discussion

We report a case of metastatic seminoma in which the testicular primary showed a pure intertubular growth pattern and radiologically mimicked a BOT. The main message of this case is diagnostic rather than comparative: in a patient with metastatic seminoma, the absence of a discrete intratesticular mass on scrotal US or mpMRI does not exclude a viable testicular primary. In our patient, the combination of testicular atrophy, parenchymal heterogeneity, calcification, and focal hypoperfused areas was initially compatible with a BOT on imaging, yet orchiectomy demonstrated diffuse viable seminoma with an exclusive intertubular pattern, associated fibro-sclerotic remodeling, and adjacent GCNIS.

Scrotal US remains the first-line testicular evaluation in metastatic seminoma, and mpMRI may be useful when ultrasound findings are negative or equivocal; however, neither modality can reliably confirm regression (10, 11). Accordingly, subtle ipsilateral testicular abnormalities—including atrophy, heterogeneity, calcifications, architectural distortion, or an ill-defined hypoperfused region—should not be interpreted as proof of regression or as evidence of an extragonadal primary. Our case therefore supports orchiectomy when metastatic seminoma is associated with subtle ipsilateral testicular abnormalities on scrotal ultrasound or mpMRI. In contrast, when the testis is entirely normal on clinical and imaging evaluation, blind orchiectomy is not routinely warranted, and the possibility of a true extragonadal germ cell tumor should be considered (3, 5–7, 10, 11).

This case also provides a plausible explanation for the imaging pitfall. Although imaging suggested BOT, pathology revealed diffuse intertubular infiltration by viable seminoma cells associated with fibro-sclerotic remodeling and GCNIS, accounting for the subtle and non-specific imaging appearance. In this growth pattern, tumor cells permeate the interstitium and surround seminiferous tubules without forming an expansile nodule, thereby escaping detection as a typical mass lesion. When tumor cellularity is low or obscured by fibrosis or inflammation, immunohistochemistry is essential to identify inconspicuous seminoma cells (12).

From a therapeutic perspective, this case also shows that once correctly identified and staged, PITS-associated metastatic seminoma can be managed according to standard seminoma principles (9). Because PITS is exceptionally rare, treatment-specific outcome data are scarce. In this setting, cisplatin-based chemotherapy remains the recommended treatment for good-risk metastatic seminoma, including three cycles of BEP.

## Conclusion

Clinically, this case supports a practical approach: when metastatic seminoma is diagnosed and any ipsilateral testicular abnormality is present, orchiectomy remains essential to secure definitive primary-site pathology and local control and to avoid misclassification as BOT or extragonadal disease.

## Patient perspective

Receiving the diagnosis was frightening, especially because the scans did not show an obvious tumor in the testicle. The medical team explained that surgery and pathology were important to confirm where the cancer started and to guide treatment. Although the process was stressful, having clear communication and a final, confirmed diagnosis helped me feel reassured and able to focus on treatment and recovery.

## Data Availability

The original contributions presented in the study are included in the article/supplementary material. Further inquiries can be directed to the corresponding author.
